# Paediatric Utilisation of Ophthalmic Antibiotics in the Ear in Aotearoa/New Zealand

**DOI:** 10.3390/children12050557

**Published:** 2025-04-25

**Authors:** Isabella Mei Yan Cheung, Tary Yin, Akilesh Gokul

**Affiliations:** 1 Department of Ophthalmology, University of Auckland, 85 Park Road, Grafton, Auckland 1023, New Zealand; akilesh.gokul@auckland.ac.nz; 2 Department of Surgery, University of Auckland, 85 Park Road, Grafton, Auckland 1023, New Zealand

**Keywords:** child, New Zealand, anti-bacterial agents, eye infections, otitis

## Abstract

Background/Objectives: Some ophthalmic antibiotics are publicly subsidised in New Zealand (NZ) for off-label use in the ear, however, this utilisation has not previously been described. This study compared the utilisation of ophthalmic chloramphenicol and ciprofloxacin in the eye and ear, among NZ children. Methods: This study involved clinical record review, and included 11,617 prescriptions of ophthalmic chloramphenicol and ciprofloxacin in 2022, for children aged five years or under in Auckland, NZ. Prescriptions of chloramphenicol and ciprofloxacin for eye and ear use were compared by: patient age, gender, ethnicity and socioeconomic deprivation, indication, community or hospital prescribing and number of repeat prescriptions. Statistical analysis was performed using Chi-squared test and multinomial regression. Results: Most ophthalmic ciprofloxacin was used in the ear (84%). In contrast, almost all chloramphenicol was used in the eye (96%). Post-operative use following tympanostomy tube insertion accounted for half of all hospital-prescribed ophthalmic ciprofloxacin used in the ear. Utilisation of chloramphenicol and ciprofloxacin in the eye and ear was similar, with more prescriptions for children aged one year and males, and most children received only one prescription. Māori and Pacific children generally received fewer prescriptions. Pacific children were more likely than Māori children to receive hospital-prescribed ophthalmic ciprofloxacin for use in the ear (adjusted OR 6.7, *p* = 0.025). Conclusions: These findings highlight the utilisation of ophthalmic ciprofloxacin in the ear in NZ children. These findings will inform decision-making in the public funding of medications, policy development in equitable medication access, and more collaborative efforts to improve antimicrobial use.

## 1. Introduction

Paediatric utilisation of ophthalmic antimicrobials is relatively high [1], and these antibiotics are often used in the management of acute bacterial conjunctivitis [2], which is highly prevalent in the paediatric population. However, serious complications are rare [2], and most cases are self-limiting [3]. Antimicrobial therapy aims to speed resolution and decrease transmission; however, evidence supporting such use is limited [3].

Antibiotic exposure drives the development of resistance, and resistant organisms have been isolated from bacterial eye swabs from children [4]. Additionally, topical antibiotic use can alter the ocular surface microbiome in children [5], and changes to the microbiome have been found in some ocular surface conditions [6].

In New Zealand (NZ), chloramphenicol and ciprofloxacin are among the most commonly used ophthalmic antibiotics [7]. Of note, these ophthalmic formulations are also publicly subsidised in NZ for off-label use in the ear. Topical antibiotics are often used in the ear in the management of conditions such as chronic suppurative otitis media [8], which is prevalent among children. However, there are concerns around the development of antimicrobial resistance and potential ototoxicity, related to antibiotic exposure in the ear [9,10].

There is limited evidence on the utilisation of ophthalmic antibiotics in the ear in NZ. As such, this study aimed to compare the use of ophthalmic chloramphenicol and ciprofloxacin between eye and ear conditions, with a focus on usage among young children. This study found the utilisation of ophthalmic ciprofloxacin in the ear to be common among NZ children.

## 2. Materials and Methods

Ethical approval for this study was obtained from the Auckland Health Research Ethics Committee (reference AH24592). Institutional approval was obtained from the Te Whatu Ora Auckland Research Review Committee (reference A+9733). As this cross-sectional study involved retrospective analysis of hospital records only, and did not involve working with human participants, informed consent and Declaration of Helsinki are not applicable for this analysis.

This study included every prescription of 0.5% chloramphenicol and 0.3% ciprofloxacin eye drops between January and December 2022, for children aged five years or under with a public hospital record, within Health NZ-Te Whatu Ora Auckland, Counties Manukau and Waitemata health districts in Auckland, NZ. This analysis included medications prescribed and dispensed in the community, and medications prescribed during a hospital visit for subsequent dispensing in the community. In-hospital medication use and hospital-supplied medications were excluded from this analysis.

The proportion of chloramphenicol and ciprofloxacin prescriptions for use in the eye and ear was quantified; site of use was ascertained from the prescribing notes. The proportion of community and hospital prescriptions was also compared between eye and ear conditions. Specific indications for chloramphenicol and ciprofloxacin use were ascertained for hospital prescriptions; as this study analysed hospital records, indications for community prescriptions were unavailable and unable to be included in this analysis.

Number of prescriptions within the study period and patient age, gender, ethnicity and socioeconomic deprivation were compared between eye and ear use for chloramphenicol and ciprofloxacin. Proportion of patients receiving one or multiple prescriptions was compared. Proportion of prescriptions by age in years, gender, ethnicity and deprivation quintile was compared. Neighborhood deprivation was based on NZDep2013 Index of Deprivation decile [11], and deciles were aggregated into equally sized quintiles. Population proportions by gender and ethnicity were obtained from the 2018 Census.

The following factors were quantified based on every prescription included in this study: proportion of prescriptions for eye and ear use, number of prescriptions within study period and patient age. Due to the large number of prescriptions and manual extraction of the following data, the following factors were quantified based on a subset of 300 randomly sampled prescriptions, for chloramphenicol for eye use and ciprofloxacin for ear use: proportion of hospital and community prescriptions, specific indication and patient gender, ethnicity and deprivation. As there were fewer than 300 prescriptions of ciprofloxacin specified for eye use, and of chloramphenicol for ear use, these factors were quantified based on every prescription included in this study.

Data and statistical analyses were performed using IBM SPSS Statistics version 29 (Armonk, NY, USA). Missing data was omitted from analysis. Chi-squared test was performed to determine significant differences in proportions of prescriptions between comparison groups. Multinomial regression was performed to determine independent and significant associations between: (1) receiving a hospital prescription and age, gender, ethnicity and deprivation; (2) receiving a prescription before one year of age and gender, ethnicity and deprivation; and (3) receiving multiple prescriptions and gender, ethnicity and deprivation. As such, potential confounders such as ethnicity and deprivation were adjusted for in this regression analysis. *p* ≤ 0.05 was considered to be statistically significant.

## 3. Results

This analysis captured 10,227 chloramphenicol and 1390 ciprofloxacin prescriptions. Notably, most ciprofloxacin was used in the ear (84%), and only 9% was for ophthalmic use (*n* = 1168 and 125 respectively). In contrast, almost all chloramphenicol was for ophthalmic use (96%), and only 0.3% was used in the ear (*n* = 9818 and 31 respectively). Site of use was not specified for 3.7% (*n* = 378) and 7% (*n* = 97) of chloramphenicol and ciprofloxacin prescriptions respectively, and these were not analysed further.

The following factors were quantified based on every prescription with specified site of use, as described above: number of repeat prescriptions and patient age. Due to the large number of prescriptions of ciprofloxacin for ear use and chloramphenicol for eye use, other factors were quantified based on subsets of 300 randomly selected prescriptions: proportion of hospital and community prescriptions, specific indication and patient gender, ethnicity and deprivation. For ciprofloxacin for eye use and chloramphenicol for ear use, quantification of these factors was based on every prescription as there were fewer than 300.

Most antibiotics were community-prescribed (Table 1); however, the proportion of hospital prescriptions was significantly larger for usage in the ear, compared to ophthalmic use (*p* < 0.001). Almost all chloramphenicol for ophthalmic use was community prescribed (98.7%).

For hospital prescriptions, a large proportion of ciprofloxacin used in the ear (50%) was attributed to post-operative use following tympanostomy tube insertion (Table 2). Otitis externa, otitis media and otorrhea also contributed to ciprofloxacin and chloramphenicol usage in the ear. Indications for ophthalmic use of ciprofloxacin included corneal ulcers, microbial keratitis and bacterial conjunctivitis, while indications for ophthalmic use of chloramphenicol included viral conjunctivitis.

Most children received only one prescription over the study period (Table 1). However, with ciprofloxacin, a larger proportion of children received multiple prescriptions for ear usage compared with ophthalmic use (29.1% and 17.4% respectively). Conversely with chloramphenicol, a larger proportion of children received multiple prescriptions for ophthalmic use, compared with ear usage. Indeed, almost every child (96.3%) receiving chloramphenicol for ear usage received only one prescription over the study period. These differences were statistically significant (*p* < 0.001).

Children aged one year accounted for the most prescriptions (Table 1); this was more marked with chloramphenicol usage in the ear (38.7%). A large proportion of chloramphenicol for ophthalmic use was also accounted for by children aged two years (24.6%). These differences were statistically significant (*p* < 0.001).

Males accounted for a larger proportion of prescriptions (Table 1), and these proportions exceeded their population proportion (51.4%). This preponderance was more marked with chloramphenicol use in the ear and ciprofloxacin use in the eye (67.7% and 65.6% respectively). These differences were statistically significant (*p* = 0.004).

The proportions of prescriptions accounted for by Māori and Pacific children were generally smaller than their population proportions of 18.1% and 27.5% respectively (Table 1). In exception, Pacific children accounted for a slightly larger proportion of chloramphenicol used in the ear (29%) than their population proportion. Among Māori children, ophthalmic usage was particularly low (5.6% for ciprofloxacin and 11% for chloramphenicol). Among Pacific children, use of ciprofloxacin in the ear and chloramphenicol in the eye was particularly low (both 8%). These differences were statistically significant (*p* < 0.001).

Proportions of prescriptions by socioeconomic deprivation quintile generally varied between 16% and 23% (Table 1). In exception, chloramphenicol usage in the ear was particularly high in the most deprived quintile (quintile 5, 38.7%), while usage was particularly low in quintile 4 (6.5%). These differences were statistically significant (*p* = 0.009).

By and large, there were no independent and statistically significant relationships between: (1) receiving a hospital prescription and age, gender, ethnicity or deprivation; (2) receiving a prescription before one year of age and gender, ethnicity or deprivation; or (3) receiving multiple prescriptions and gender, ethnicity or deprivation (all *p* ≥ 0.05). In exception, for ciprofloxacin used in the ear, Pacific ethnicity was independently associated with an increased likelihood of receiving a hospital prescription, compared with Māori ethnicity (adjusted odds ratio (OR) 6.7, 95% confidence interval (CI) 1.3–35.4, *p* = 0.025). Adjusted OR was adjusted for all other factors i.e., deprivation as a potential confounder, as well as age and gender. Unadjusted OR was 0.16 (95% CI 0.030–0.81, *p* = 0.028). There was no statistically significant difference between Māori and non-Māori/non-Pacific children (*p* = 0.059).

## 4. Discussion

These findings suggest that ophthalmic ciprofloxacin is commonly used in the ear among NZ children. Although ototopical ciprofloxacin/dexamethasone is available in NZ and commonly used for a number of ear conditions [12], the use of ophthalmic ciprofloxacin in the ear could partly reflect the lack of subsidisation for ototopical ciprofloxacin/dexamethasone (as a community pharmaceutical), while ophthalmic ciprofloxacin is subsidised for use in the ear and the treatment of chronic suppurative otitis media.

Topical ciprofloxacin is often used in patients with tympanic membrane perforation or a tympanostomy tube [13,14], as medication may reach the inner ear and ciprofloxacin has a lower risk of ototoxicity [15]. This could contribute to the use of ophthalmic ciprofloxacin in the ear, rather than another ototopical anti-infective such as framycetin/gramicidin [10], the main therapeutic option available and funded in NZ. Potential ototoxicity could also explicate the low usage of ophthalmic chloramphenicol in the ear described in this study [10], despite this ophthalmic medication also being funded for use in the ear.

These findings suggest that ophthalmic ciprofloxacin (for both eye and ear use) is often hospital-prescribed, compared with chloramphenicol which is almost exclusively community-prescribed and used in the eye. Chloramphenicol usage could reflect the frequent and empirical use of anti-infectives in common and relatively mild ocular conditions, particularly in primary care [16]. In contrast, ciprofloxacin is typically used for more serious ocular infections such as corneal ulcers and microbial keratitis, which are common presentations to acute tertiary eye services [17,18]. These findings also suggest that ophthalmic ciprofloxacin is also used in the ear following tympanostomy tube insertion, and that this medication is prescribed by hospitals for dispensing and subsidisation as a community pharmaceutical for this usage. Interestingly, ototopical ciprofloxacin/dexamethasone is subsidised as a hospital medication and can be supplied by hospitals for patient use following a hospital visit. This could potentially reflect the utilisation of different sources of public funding in the provision of ciprofloxacin for this usage.

Topical antibiotics are important in the treatment of both eye and ear conditions. Antibiotics are effective in the treatment of serious bacterial eye infections such as bacterial keratitis, in which delayed treatment can lead to significant corneal damage and vision loss, necessitating a corneal transplant [19]. Antibiotics are also effective in the treatment of some infectious conditions of the ear common in children, such as chronic suppurative otitis media, which left untreated can lead to tympanic membrane perforation, hearing loss and life-long sequalae [20]. As such, topical antibiotics are important when necessary in the treatment of some eye and ear conditions, some of which are common in children.

However, reducing the routine use and overuse of antimicrobials is also a wide-reaching and important issue. In the eye, topical antibiotics are often used unnecessarily in some conditions common in children, which usually resolve spontaneously without complications. For instance, anti-infectives are often used in the management of ocular conditions such as acute bacterial conjunctivitis [2]; however most cases are self-limiting and serious complications are rare [2,3]. Antimicrobial therapy aims to decrease transmission and speed resolution; however, evidence supporting such use is limited [3]. Anti-infectives are also often used in ocular adenoviral infections [18], which are also highly prevalent among children. However, the evidence again does not suggest any benefit, as the risk of secondary bacterial infection is negligible [21]. Furthermore, antibiotics are often used unnecessarily in allergic conjunctivitis (again common in children) as this is challenging to differentiate from infective conjunctivitis [22]. Notably, due to concerns around resistance, eye care professionals in the tertiary setting have increasingly been reserving the use of ophthalmic ciprofloxacin for the treatment of bacterial keratitis [18]. However, these findings suggest that ophthalmic ciprofloxacin is also used in the ear, in both the community and hospital settings. Topical antibiotics are often used in the ear in the management of conditions common in children such as chronic suppurative otitis media [8]. Tympanostomy tube insertion and post-operative otorrhea are also very common among children [8,23], and topical anti-infectives are often used prophylactically [24]. However, alternative methods which use less or no antibiotics for reducing tympanostomy tube complications have recently been described [23]. As such, while topical antibiotics are important when necessary in the treatment of some eye and ear conditions, opportunities and alternative approaches to reduce unnecessary use could be explored. In the eye, better diagnosis and differentiation between serious conditions which need timely antibiotic treatment, and common but mild conditions in which antibiotics are ineffective, remain key. In the ear, the efficacy of multiple saline washouts and a single application of antibiotic during tympanostomy tube insertion has previously been described, and could be further explored [23].

Antimicrobial resistance in the context of infectious conditions in the eye and ear, and whether this could potentially impact children disproportionately, could also be explored. Due to the particularly high prevalence of these conditions among children, the utilisation of antibiotics in the eye and ear is particularly high in the paediatric population, which likely contributes to the development of resistance. Indeed, resistant organisms have been found in paediatric isolates from the eye and ear [4,25,26,27]. Furthermore, antibiotic use in some of these conditions is often empirical with broad spectrum therapies [28,29], further increasing antimicrobial exposure and their potentially suboptimal use. Infections caused by resistant organisms are more likely to result in treatment failure, increasing the likelihood of more severe and prolonged infection and structural damage. In the context of infectious conditions in the eye and ear in the paediatric population, it is possible this can lead to visual and hearing impairment, which can affect developmental and educational progress in cases of long-term sensory loss [30,31]. Further antibiotic utilisation and susceptibility studies, and improved microbial and susceptibility testing, treatment guidelines and antimicrobial stewardship strategies—all focused on the paediatric population—will help address antimicrobial resistance in children.

Māori and Pacific children generally accounted for a smaller proportion of prescriptions (compared with population proportions) of ophthalmic antibiotics for both eye and ear use. This is particularly concerning as the incidence of some common indications, such as otitis media, may be higher among these populations [32]. It is possible that low utilisation among Māori and Pacific children could reflect poorer healthcare access [33], potentially contributing to under-treatment of these conditions. Indeed, tympanostomy tube insertion rates may be lower among Māori and Pacific children, particularly those residing in areas of higher socioeconomic deprivation [32]. This is particularly concerning as higher proportions of Māori and Pacific live in more deprived areas [34], and the development of otitis media and keratitis has also been linked to deprivation [32,34]. Regarding severity of disease, the literature on whether particular sociodemographic groups in NZ experience more severe disease in infectious conditions of the eye and ear is limited. However, it has been reported that Indigenous Australians experience more severe disease in otitis media [35]. Socioeconomic deprivation has also been linked to receiving treatment for severe complications of otitis media in the United States [36]. Patients of African-American descent, and those with public insurance and housing insecurity, are at higher risk of longer hospitalisation following presentation with a corneal ulcer [37]. Neighborhood deprivation has also been associated with worse visual acuity in patients with microbial keratitis in the United States [38]. Whether particular sociodemographic groups in NZ experience more severe disease in infectious conditions of the eye and ear needs to be better described, to inform health equity programmes across NZ.

Equitable antibiotic utilisation is an important and complex issue. Many studies report lower antibiotic utilisation among many minoritised sociodemographic groups worldwide. Ethnic and racial identity, and migration, employment and insurance status have been linked to antibiotic utilisation in some analyses, although contrasting findings have also been reported by some studies [39,40,41]. This is particularly concerning as infectious disease burden is often higher among minoritised groups [40,42,43,44]. Improving the equity of antibiotic utilisation is complex, as many sociodemographic factors intersect and impact both healthcare-seeking and healthcare-providing behaviours. Multidimensional approaches are needed; these could include addressing economic barriers to access and challenges related to healthcare delivery, and health promotion programmes tailored for specific minoritised communities. Antimicrobial stewardship policies should also be developed through an equity lens, to ensure equal health outcomes for all children while encouraging appropriate medication use.

This analysis had some limitations. Firstly, the parameters of this analysis resulted in a relatively small number of prescriptions of chloramphenicol for ear use and ciprofloxacin for eye use being captured. As such, the strength of these findings may be lower. Although such usage is less common, further analyses of such usage will help add to the evidence base on antibiotic utilisation in NZ. Secondly, ophthalmic chloramphenicol can also be provided by pharmacists without a prescription in NZ, under certain conditions. These dispensings were excluded from this analysis, as the availability of such data is very limited in NZ. As such, ophthalmic chloramphenicol usage may be under-represented in this analysis. However, ophthalmic chloramphenicol can only be provided by pharmacists for patients aged over two years, and its usage is highest in children aged under two years. As such, the influence of pharmacist dispensings on this analysis is anticipated to be minimal. Thirdly, some factors were quantified based on subsets of prescriptions, rather than every prescription. However, as these subsets included 300 randomly selected prescriptions, the generalisability of these findings is considered to be adequate.

In conclusion, this study highlighted the utilisation of ophthalmic ciprofloxacin in the ear in NZ children, which was previously undescribed, and will help inform policy decision-making in the public funding of both ophthalmic and ototopical ciprofloxacin. These findings also highlight the need for more collaborative efforts to improve the appropriate use of ophthalmic ciprofloxacin, which also need to be balanced with adequate and equitable access to this medication, particularly for Māori and Pacific children. These findings will help support initiatives to improve access, uptake and equity of ear health services in NZ.

## Figures and Tables

**Table 1:** Patient demographics and prescribing-related factors for prescriptions of chloramphenicol and ciprofloxacin for eye and ear use.

		Ciprofloxacin		Chloramphenicol		Total
		Ear	Eye	Ear	Eye	
Patient age ^†^ (% (n) of prescriptions)	Under 1 year	8.8 (103)	13.6 (17)	9.6 (3)	8.1 (795)	8.2 (918)
	1 year	27.2 (318)	27.2 (34)	38.7 (12)	26.2 (2572)	26.4 (2936)
	2 years	17.6 (206)	11.2 (14)	19.4 (6)	24.6 (2415)	23.7 (2641)
	3 years	13.7 (160)	14.4 (18)	12.9 (4)	18.9 (1855)	18.3 (2037)
	4 years	17.8 (208)	15.2 (19)	12.9 (4)	16.3 (1600)	16.4 (1831)
	5 years	14.8 (173)	18.4 (23)	6.4 (2)	5.9 (581)	7.0 (779)
	Unknown	0	0	0	0	0
Patient gender ^‡^ (% (n) of prescriptions)	Male	54 (162)	65.6 (82)	67.7 (21)	54.7 (164)	56.7 (429)
	Female	46 (138)	34.4 (43)	32.3 (10)	45.3 (136)	43.3 (327)
	Unknown	0	0	0	0	0
Patient ethnicity ^‡^ (% (n) of prescriptions)	Māori	9.7 (29)	5.6 (7)	16.1 (5)	11 (33)	9.8 (74)
	Pacific Peoples	8.0 (24)	12.0 (15)	29 (9)	8.0 (24)	9.5 (72)
	Non-Māori/ Non-Pacific	75.0 (225)	77.6 (97)	54.8 (17)	81 (243)	77.0 (582)
	Unknown	7.3 (22)	4.8 (6)	0	0	3.7 (28)
Socioeconomic deprivation quintile ^‡^ (% (n) of prescriptions)	1 (least deprived)	23.3 (70)	16 (20)	19.3 (6)	22.3 (67)	21.6 (163)
	2	22.0 (66)	20 (25)	12.9 (4)	18.3 (55)	19.8 (150)
	3	16.7 (50)	24.8 (31)	22.6 (7)	25.7 (77)	21.8 (165)
	4	17.7 (53)	20.8 (26)	6.5 (2)	18.6 (56)	18.1 (137)
	5 (most deprived)	19.3 (58)	18.4 (23)	38.7 (12)	12.7 (38)	17.3 (131)
	Unknown	1.0 (3)	0 (0)	0 (0)	2.3 (7)	1.3 (10)
Community/hospital prescribing ^‡^ (% (n) of prescriptions)	Community	78.7 (236)	88.8 (111)	80.6 (25)	98.7 (296)	88.4 (668)
	Hospital	21.3 (64)	11.2 (14)	19.4 (6)	1.3 (4)	11.6 (88)
	Unknown	0	0	0	0	0
Single/multiple prescriptions ^†^ (% (n) of patients)	1 prescription	70.9 (697)	82.6 (95)	96.3 (26)	79.9 (6215)	79.0 (7033)
	2 or more prescriptions	29.1 (286)	17.4 (20)	3.7 (1)	20.1 (1561)	21.0 (1868)
	Unknown	0	0	0	0	0

^†^ based on every prescription. ^‡^ based on subset of 300 randomly selected prescriptions for ciprofloxacin for ear use and chloramphenicol for eye use, and every prescription of ciprofloxacin for eye use and chloramphenicol for ear use.

**Table 2:** Primary indications for hospital prescriptions of chloramphenicol and ciprofloxacin for eye and ear use.

Primary Indication	% (*n*)
Ciprofloxacin—ear use	
Tympanostomy tube insertion	50 (32)
Otitis media	18.8 (12)
Otorrhea	10.9 (7)
Otitis externa	6.3 (4)
Other	14 (9)
Chloramphenicol—ear use	
Otitis externa	33.3 (2)
Otitis media	33.3 (2)
Otorrhea	33.3 (2)
Ciprofloxacin—ophthalmic use	
Corneal ulcer	21.4 (3)
Conjunctivitis—bacterial	21.4 (3)
Keratitis—unspecified	21.4 (3)
Keratitis—microbial	14.3 (2)
Other	21.4 (3)
Chloramphenicol—ophthalmic use	
Conjunctivitis—viral	50 (2)
Other	50 (2)

## Data Availability

The datasets presented in this article are not readily available as confidential patient data from hospital records were analysed in this study.
